# Depletion of perivascular macrophages delays ALS disease progression by ameliorating blood-spinal cord barrier impairment in SOD1^G93A^ mice

**DOI:** 10.3389/fncel.2023.1291673

**Published:** 2023-11-24

**Authors:** Kazuki Adachi, Kota Miyata, Yukino Chida, Mikako Hirose, Yuta Morisaki, Koji Yamanaka, Hidemi Misawa

**Affiliations:** ^1^Division of Pharmacology, Faculty of Pharmacy, Keio University, Tokyo, Japan; ^2^Department of Neuroscience and Pathobiology, Research Institute of Environmental Medicine, Nagoya University, Nagoya, Japan

**Keywords:** amyotrophic lateral sclerosis, SOD1^G93A^ mouse, perivascular macrophage, clodronate liposomes, blood-spinal cord barrier, extracellular matrix

## Abstract

Amyotrophic lateral sclerosis (ALS) is a fatal motor neuron disease in which non-cell-autonomous processes have been proposed as its cause. Non-neuronal cells that constitute the environment around motor neurons are known to mediate the pathogenesis of ALS. Perivascular macrophages (PVM) are immune cells that reside between the blood vessels of the central nervous system and the brain parenchyma; PVM are components of the neurovascular unit and regulate the integrity of the blood-spinal cord barrier (BSCB). However, it is not known whether regulation of BSCB function by PVM is involved in the pathogenesis of ALS. Here, we used SOD1^G93A^ mice to investigate whether PVM is involved in the pathogenesis of ALS. Immunostaining revealed that the number of PVM was increased during the disease progression of ALS in the spinal cord. We also found that both anti-inflammatory Lyve1^+^ PVM and pro-inflammatory MHCII^+^ PVM subtypes were increased in SOD1^G93A^ mice, and that subtype heterogeneity was shifted toward MHCII^+^ PVM compared to wild-type (WT) mice. Then we depleted PVM selectively and continuously in SOD1^G93A^ mice by repeated injection of clodronate liposomes into the cerebrospinal fluid and assessed motor neuron number, neurological score, and survival. Results showed that PVM depletion prevented the loss of motoneurons, slowed disease progression, and prolonged survival. Further histological analysis showed that PVM depletion prevents BSCB collapse by ameliorating the reduction of extracellular matrix proteins necessary for the maintenance of barrier function. These results indicate that PVM are involved in the pathogenesis of ALS, as PVM degrades the extracellular matrix and reduces BSCB function, which may affect motor neuron loss and disease progression. Targeting PVM interventions may represent a novel ALS therapeutic strategy.

## 1 Introduction

Amyotrophic lateral sclerosis (ALS) is a neurodegenerative disease that affects motor neurons, leading to muscle weakness, progressive paralysis, and death. While most ALS patients are sporadic, approximately 10% are familial and 20% of familial ALS patients have mutations in Cu/Zn-superoxide dismutase 1 (SOD1). Transgenic mice carrying the human mutant SOD1^G93A^ gene recapitulate the clinical symptoms and pathological features of ALS patients and are widely used as animal models of ALS.

A non-cell-autonomous process has been proposed as the cause of ALS, in which non-neuronal cells that constitute the environment around motor neurons mediate the pathogenesis of the disease (Van Harten et al., 2021). The diverse cell populations around motor neurons, including endothelial cells, pericytes, astrocytes, and microglia, constitute a structural and functional neurovascular unit (NVU) that maintains central nervous system (CNS) homeostasis (Liebner et al., 2018). Disruption of the blood-brain barrier (BBB) and blood-spinal cord barrier (BSCB), which function to effectively regulate the transfer of substances from the periphery to the CNS, has been observed in ALS mouse models (Zhong et al., 2008; Yoshikawa et al., 2022) and patients (Garbuzova-Davis et al., 2012). Furthermore, the extracellular matrix (ECM) of the vascular basement membrane, including laminin, collagen IV, nidogen, HSPGs (perlecan and agrin), and fibronectin, is an important regulator of BBB and BSCB (Reed et al., 2019), and decreased laminin expression in the spinal cord of SOD1^G93A^ mice was reported (Garbuzova-Davis et al., 2007).

Macrophages that are resident in the CNS border region are known as CNS-associated macrophages and are classified as perivascular macrophages (PVM), meningeal macrophages, and choroid plexus macrophages. These macrophages are thought to play important roles in immune responses at the CNS border under physiological and pathological conditions, but studies of their detailed roles have only recently begun (Prinz et al., 2021). Perivascular macrophages (PVM) are innate immune cells that reside in the cerebrospinal fluid (CSF)-filled perivascular space (Virchow-Robin space) between the glial limitans of the brain parenchyma and the abluminal side vascular basement membrane (Yang et al., 2019). PVM are defined as a distinct population that is different anatomically and genetically from microglia that are resident in the parenchyma (Goldmann et al., 2016). PVM are a component of NVUs and maintain the integrity of the BBB under physiological conditions (Willis et al., 2007). However, under pathological conditions, PVM has been shown to disrupt the BBB (Pedragosa et al., 2018; Santisteban et al., 2020). Recent studies have shown that PVM, like peripheral macrophages, contribute to ECM degradation (Drieu et al., 2022). However, no studies have so far examined the involvement of PVM in the pathogenesis and progression of ALS.

In this study, we investigated PVM during disease progression in a mouse model of ALS. We selectively depleted PVM by intracisternal injection of clodronate liposomes (CLO) into mice and examined the involvement of PVM in ALS pathological progression, focusing on the regulation of BBB/BSCB integrity.

## 2 Materials and methods

### 2.1 Mice

Transgenic mice carrying the human mutant SOD1^G93A^ gene [B6.Cg-Tg(SOD1*G93A)1Gur/J] were purchased from Jackson Laboratory and maintained on a C57BL/6 genetic background. All experiments were reviewed and approved by the Keio University Animal Care and Use Committee, and care was taken to minimize suffering and limit the number of animals used. We used females for the experiments.

### 2.2 Intracisternal injection

#### 2.2.1 Ovalbumin Alexa Fluor*™* 555 conjugate injection

140-day-old SOD1^G93A^ mice and matching wild-type (WT) mice were anesthetized by subcutaneous injection of MMB mixture (0.3 mg kg^–1^ medetomidine hydrochloride, 4 mg kg^–1^ midazolam, and 5 mg kg^–1^ butorphanol) and exposed the cisterna magna. Then, mice were secured in a stereotaxic frame and the head was tilted downward. 1% fluorescent OVA (OVA-555: Ovalbumin Alexa Fluor*™* 555 conjugate; Thermo, Waltham, MA, USA) in an artificial CSF (126 mM NaCl, 3 mM KCl, 1.25 mM NaH_2_PO_4_, 2 mM MgSO_4_, 2 mM CaCl_2_, 10 mM D-glucose, and 26 mM NaHCO_3_) was filled into PE10 tube (Polyethylene Tubing 0.61 mm OD x0.28 mm ID) with a 30G needle, then the needle was inserted into cisterna magna. The needle was fixed in place by Aron Alpha^®^ A (Daiichi Sankyo) before injection. 10 μl of OVA-555 was injected at a rate of 1 μl/min using a syringe pump. To prevent backflow, the syringe was left in place for 5 min after injection. The tube was cut and sealed at its end.

#### 2.2.2 Clodronate liposomes injection into SOD1^G93A^ or WT mice

10 μl of phosphate-buffered saline (PBS)-liposomes or clodronate-liposomes (1:1 mixture of MKV100 and MKV300; CosmoBio, Tokyo, Japan) were injected into mice with a 27G needle connected to PE20 tube (Polyethylene Tubing 1.09 mm OD x 0.38 mm ID) as described above. Before the tube was cut and sealed, the needle was fixed in place with cement. Atipamezole (3 mg kg^–1^) was administered subcutaneously at the end of surgery. For the second injection, mice were anesthetized by subcutaneous injection of MMB mixture, then a new tube with the needle was connected to the fixed tube and injected.

### 2.3 Immunohistochemistry

Mice were deeply anesthetized with isoflurane and then perfused via the aortic cone with PBS, followed by 4% paraformaldehyde (PFA) in 0.1 M phosphate buffer at pH 7.4 (PB). The lumbar spinal cords were post-fixed in 4% PFA for 24 h, followed by immersion in 20% sucrose in 0.1 M PB overnight. Sections were sliced at 40 μm using a cryostat. Free-floating frozen sections were treated with Histo VT one (Nacalai Tesque, Kyoto, Japan) for antigen retrieval as needed, then blocked with 1% skim milk, 2% bovine serum albumin, and 3% normal donkey serum, in PBS containing 0.1% Triton X-100 (PBS-T). Sections were incubated with primary antibodies overnight at 4^°^C followed by an incubation with appropriate Alexa Fluor-conjugated secondary antibodies (diluted 1:500 in the blocking buffer as above; Thermo, Waltham, MA, USA) for 1 h, at room temperature and wet-mounted in Fluoromount-G (Southern Biotechnology, Birmingham, AL, USA). The primary antibodies and dilutions used were as follows: rat anti-CD206 (1:200, MCA2235, Bio-Rad, Hercules, CA, USA), goat anti-CD31 (1:300, AF3628, R&D Systems, Minneapolis, MN, USA), rabbit anti-Iba1 (1:1,000, 019-19741, Wako, Osaka, Japan), Alexa Fluor 488 conjugated rat anti-CD206 (1:50, MCA2235A488T, Bio-Rad, Hercules, CA, USA), rabbit anti-Lyve1 (1:200, ab14917, Abcam, Cambridge, UK), rat anti-I-A/I-E (MHCII; 1:200, 107602, BioLegend, San Diego, CA, USA), rabbit anti-choline acetyltransferase (ChAT) (1:500) (Ichikawa et al., 1997), mouse anti-GFAP (1:1,000, G3893, Sigma, St. Louis, MO, USA), rabbit anti-laminin (1:500, ab7463, Abcam, Cambridge, UK), rabbit anti-collagen IV (1:1,000, LSL-LB-1403, CosmoBio, Tokyo, Japan). Four sections per animal were imaged (one image per section) with an Olympus FV3000 confocal microscope system (GFAP^+^ area: 20×, anterior region. Others: 10×, tiling imaging to capture the entire section), and quantitative analyses of imaging measurements were performed using Fiji (ImageJ).

### 2.4 Prussian blue staining

PFA-fixed frozen lumbar sections (described above) were incubated in a 5% potassium ferrocyanide and 5% hydrochloric acid solution (1:1 working solution) at RT for 30 min. The sections were washed and then counterstained with eosin. Four sections per animal were imaged (one image per section) with a KEYENCE BZ-X710 microscope (20×), and the number of hemosiderin-positive spots (blue spots) in the anterior region was counted. The average number of Prussian blue-positive deposits per section was then calculated for analysis.

### 2.5 Neurological score and survival assessment

Neurological score was assessed three times a week as described in the Jackson Laboratory’s strain manual (Working with ALS Mice: guidelines for preclinical testing and colony management from the Jackson Laboratory)^1^ : 0 = full extension of hindlimbs away from lateral midline when the mouse is suspended by its tail; 1 = collapse or partial collapse of leg extension toward the lateral midline (weakness) or trembling of hindlimbs during tail suspension; 2 = during walking any part of the foot is dragging along the table; 3 = rigid paralysis or minimal joint movement, foot not being used for generating forward motion; 4 = mouse cannot right itself within 30 s from either side. For the data analysis, dead mice were included in score 4. The survival endpoint was defined as when the score of “4” was reached.

### 2.6 Statistical analysis

All data are presented as the mean ± SEM. Statistical analyses were carried out using GraphPad Prism 8 software. Statistical significance was tested with the Student’s *t*-test, one-way ANOVA with Tukey’s multiple comparisons test, or two-way ANOVA with Sidak’s multiple comparisons test. Log-rank test was used for mouse survival analysis. Differences were considered statistically significant when *P*-values were less than 0.05.

## 3 Results

### 3.1 Identification of PVM in SOD1^G93A^ mice

**FIGURE 1:**
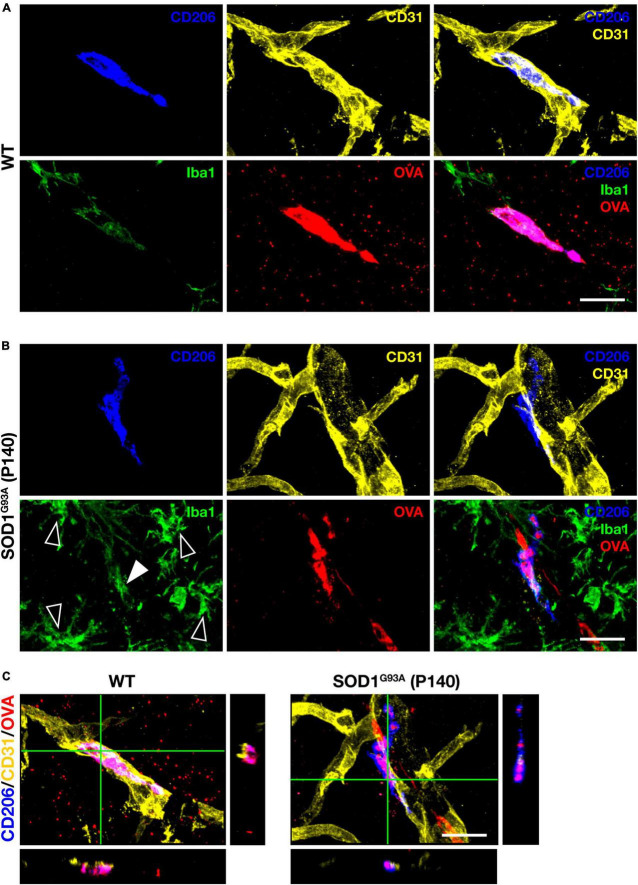
PVM characterization. Representative images of lumbar spinal cord sections showing CD206, CD31, Iba1 staining, and intracisternal injected fluorescently labeled OVA. **(A)** 140-day-old control WT mice (Scale bar = 20 μm). **(B)** Late symptomatic (P140) SOD1^G93A^ mice. White filled and blank white arrowheads indicate low levels of Iba1 and high levels of Iba1 (microglia), respectively (Scale bar = 20 μm). **(C)** Orthogonal images of WT mice and late symptomatic (P140) SOD1^G93A^ mice (Scale bar = 20μm).

Perivascular macrophages (PVM) are defined as myeloid cells that localize along blood vessels, express the mannose receptor CD206 and the macrophage marker Iba1, and can take up proteins administered into the CSF (Carare et al., 2008; Goldmann et al., 2016). We first injected fluorescently labeled tracer protein ovalbumin (OVA) into the cisterna magna, followed by immunostaining for CD206, Iba1, and the vascular endothelial cell marker CD31, and confirmed the existence of PVM in the lumbar spinal cord of WT mice according to the above definition (Figures 1A, C). We next confirmed PVM existence in 140-day-old (a late symptomatic stage) SOD1^G93A^ mice. Although the expression levels of Iba1 in PVM were weaker than those in the proliferating microglia associated with ALS pathological progression, we were able to identify PVM as CD206-positive perivascular cells in SOD1^G93A^ mice as well as in WT mice (Figures 1B, C).

### 3.2 Increase in the number of PVM and changes in their heterogeneity during the disease progression in SOD1^G93A^ mice

**FIGURE 2:**
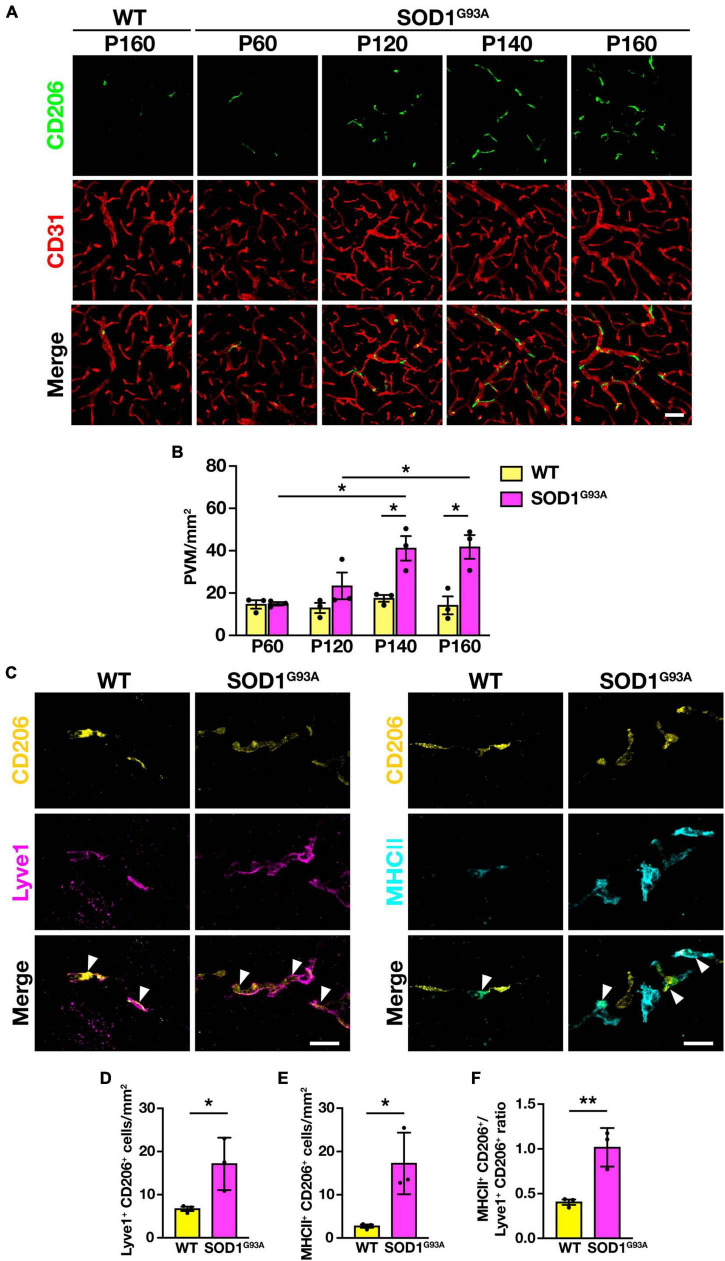
Increase in PVM number and change in subtype heterogeneity with disease progression in SOD1^G93A^ mice. **(A)** Representative images of lumbar spinal cord sections showing CD206 and CD31 staining (Scale bar = 50 μm). **(B)** Quantification of PVM (*n* = 3 per group, two-way ANOVA; genotype effect, *p* < 0.05; age effect, *p* = 0.08; genotype × age effect, *p* < 0.05, with Sidak’s multiple comparisons test. *p < 0.05). **(C)** Representative images of lumbar spinal cord sections of WT mice and end-stage (P160) SOD1^G93A^ mice showing CD206, Lyve1 and MHCII staining. Arrowheads indicate Lyve1^+^ PVM or MHCII^+^ PVM (Scale bar = 20 μm). **(D)** Quantification of Lyve1^+^ PVM. **(E)** Quantification of MHCII^+^ PVM. **(F)** Quantification of MHCII^+^ PVM/Lyve1^+^ PVM ratio. For **(D–F)**, *n* = 3 per group, Student’s *t*-test. *p < 0.05, **p < 0.01. Data are presented as the mean ± SEM.

To investigate the changes in PVM during ALS disease progression, we counted the number of PVM in the lumbar spinal cord of SOD1^G93A^ mice at P60 (a pre-symptomatic stage), P120 (an early symptomatic stage), P140 (a late symptomatic stage), and P160 (an end-stage), by immunostaining for CD206 and CD31. The number of PVM in SOD1^G93A^ mice was comparable to WT mice at P60 but increased with disease progression from P120 onward (Figures 2A, B).

As previously reported, PVM can be divided into two subtypes: anti-inflammatory (M2 macrophage-like) Lyve1^+^ PVM and pro-inflammatory (M1 macrophage-like) MHCII^+^ PVM (Jeong et al., 2022). We counted the number of both subtypes in the lumbar spinal cord of end-stage (P160) SOD1^G93A^ mice, in which CD206^+^ PVM were significantly increased, and found that both Lyve1^+^ PVM and MHCII^+^ PVM were increased compared to WT mice (Figures 2C–E). Furthermore, the ratio of MHCII^+^ PVM to Lyve1^+^ PVM was higher in end-stage SOD1^G93A^ mice compared to WT mice (Figure 2F). These results indicate that PVM in WT mice mostly have an anti-inflammatory Lyve1^+^ phenotype, whereas PVM are shifting toward a pro-inflammatory phenotype with a greater MHCII^+^ positivity in SOD1^G93A^ mice. These findings suggest that there are quantitative and qualitative changes in the PVM in SOD1^G93A^ mice during ALS disease progression.

### 3.3 Repetitive intracisternal injection of clodronate liposomes can sustainably deplete PVM

**FIGURE 3:**
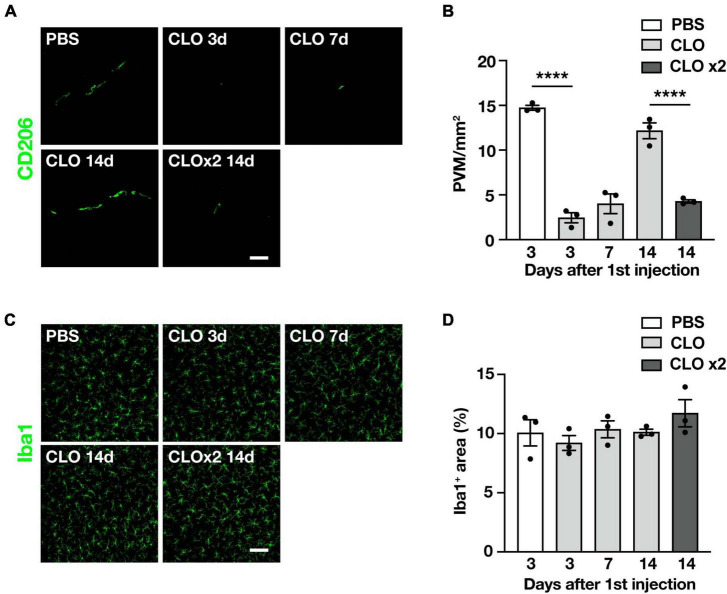
Sustained and selective PVM depletion by repetitive CLO intracisternal injection into WT mice. **(A)** Representative images of lumbar spinal cord sections showing CD206 staining (Scale bar = 50 μm). **(B)** Quantification of CD206^+^ PVM (*n* = 3 per group, one-way ANOVA; treatment effect, *p* < 0.0001, with Tukey’s multiple comparisons test. ****p < 0.0001). **(C)** Representative images of lumbar spinal cord sections showing Iba1 staining (Scale bar = 100 μm). **(D)** Quantification of Iba1^+^ area (*n* = 3 per group, one-way ANOVA; treatment effect, *p* = 0.38, with Tukey’s multiple comparisons test). Data are presented as the mean ± SEM.

Since we observed quantitative and qualitative changes in PVM in SOD1^G93A^ mice, we asked whether PVM contribute to ALS disease progression. To this end, we used clodronate liposomes (CLO) as a reagent to deplete PVM. Depletion of PVM by CLO injection into the CSF via the cisterna magna is a well-established method (Drieu et al., 2022; Lin et al., 2023); CLO are phagocytosed by macrophages and induce their apoptotic cell death (Van Rooijen et al., 1996). Compared to control liposomes (encapsulated PBS), CLO injection induced depletion of PVM in the lumbar spinal cord at 3 and 7 days after injection, but the number of PVM was restored at 14 days after injection (Figures 3A, B). The transient depletion of PVM by CLO treatment and their recovery was also reported (Polfliet et al., 2001). Considering that the ALS disease progression in SOD1^G93A^ mice is a long-term process taking 2–3 months, transient depletion with a single dose of CLO is not enough. Therefore, we repeated the injection of CLO 1 week after the first dose and confirmed that the depletion was sustained (Figures 3A, B). The number of microglia in the lumbar spinal cord was not altered by CLO injection (Figures 3C, D).

### 3.4 Sustained PVM depletion by CLO delays disease progression and prolongs survival in SOD1^G93A^ mice

**FIGURE 4:**
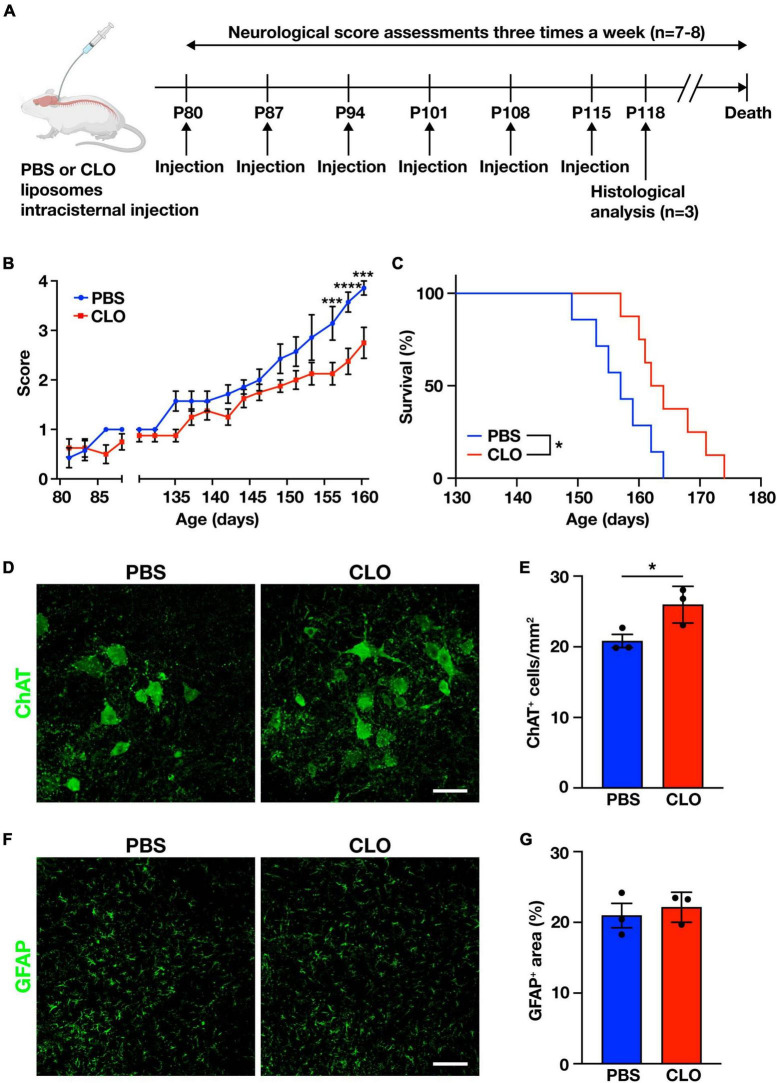
Amelioration of ALS pathology by sustained PVM depletion with CLO injection. **(A)** Experimental schematic. **(B)** Neurological mean score (*n* = 7–8 per group, two-way ANOVA; treatment effect, *p* < 0.05; age effect, *p* < 0.0001; treatment × age effect, *p* < 0.0001, with Sidak’s multiple comparisons test. ***p < 0.001, ****p < 0.0001). **(C)** Kaplan-Meier plot showing survival time (*n* = 7–8 per group, log-rank test. *p < 0.05). **(D)** Representative images of lumbar spinal cord sections showing ChAT staining (Scale bar = 50 μm). **(E)** Quantification of ChAT^+^ motor neurons (*n* = 3 per group, Student’s *t*-test. *p < 0.05). **(F)** Representative images of lumbar spinal cord sections showing GFAP staining (Scale bar = 100 μm). **(G)** Quantification of GFAP^+^ area (*n* = 3 per group, Student’s *t*-test). Data are presented as the mean ± SEM.

To determine whether PVM are involved in the disease progression, we used repeated CLO intracisternal injections as described above to sustainably deplete PVM in SOD1^G93A^ mice. PBS-liposomes or CLO-liposomes were injected once a week for a total of six times, beginning from the age of 80 days (before the disease onset), and we assessed neurological scores characterizing the severity of the disease (measured three times a week) and the total survival length (Figure 4A). The injections were limited to six times because the repeated intracisternal injection into SOD1^G93A^ mice at advanced disease stages was too invasive. Indeed, several of SOD1^G93A^ mice at the late stage of the disease were incapable of recovering from anesthesia after the intracisternal injection. Under our adopted administration scheme, the neurological scores of CLO-treated mice were significantly less symptomatic in the late symptom stage compared to PBS-treated mice (Figure 4B). Survival was also significantly prolonged in CLO-treated mice compared to PBS-treated mice (157 ± 2 days and 164.6 ± 2.1 days, PBS; *n* = 7 and CLO; *n* = 8, respectively) (Figure 4C). Furthermore, histological analysis in the lumbar spinal cord 3 days after the sixth injection (corresponding to the early symptomatic stage) showed that CLO treatment preserved more choline acetyltransferase (ChAT)-positive motor neurons (Figures 4D, E). We also evaluated astrogliosis, a hallmark of ALS, by immunostaining for GFAP, and found no significant difference between PBS-treated mice and CLO-treated mice (Figures 4F, G). These results suggest that sustained PVM depletion prevents motor neuron loss, slows disease progression, and ameliorates the pathogenesis of ALS.

### 3.5 Sustained depletion of PVM by CLO ameliorates extracellular matrix dysregulation and delays the progression of BSCB abnormalities in SOD1^G93A^ mice

**FIGURE 5:**
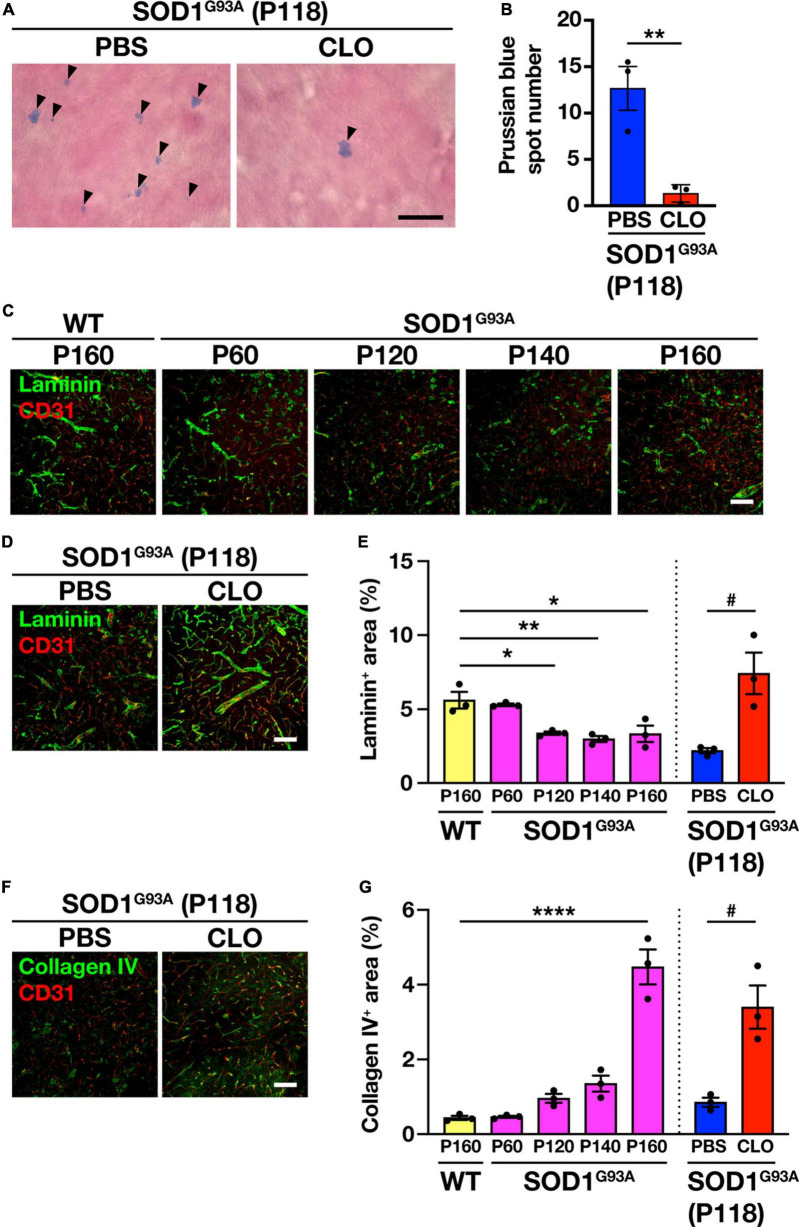
Restoration of BSCB integrity and improvement of extracellular matrix protein expression by sustained PVM depletion. **(A)** Representative images of lumbar spinal cord sections showing Prussian blue staining. Arrowheads indicate Prussian blue spots (Scale bar = 10 μm). **(B)** Quantification of Prussian blue spot numbers (*n* = 3 per group, Student’s *t*-test. **p < 0.01). **(C,D)** Representative images of lumbar spinal cord sections showing laminin and CD31 staining (Scale bar = 100 μm). **(E)** Quantification of laminin^+^ area (*n* = 3 per group. For WT and SOD1^G93A^ mice at each age, one-way ANOVA; age effect, *p* < 0.01, with Sidak’s multiple comparisons test. For PBS and CLO, Student’s *t*-test. *p < 0.05, **p < 0.01, ^#^*p* < 0.05). **(F)** Representative images of lumbar spinal cord sections showing collagen IV and CD31 staining (Scale bar = 100 μm). **(G)** Quantification of collagen IV^+^ area (*n* = 3 per group. For WT and SOD1^G93A^ mice at each age, one-way ANOVA; age effect, *p* < 0.0001, with Sidak’s multiple comparisons test. For PBS and CLO, Student’s *t*-test. ****p < 0.0001, ^#^*p* < 0.05). Data are presented as the mean ± SEM.

Since PVM depletion has been shown to ameliorate ALS pathology as described above, we sought to identify the mechanism of this amelioration. Since BSCB destruction is a pathological hallmark of ALS (Zhong et al., 2008; Yoshikawa et al., 2022) and PVM depletion restores BBB/BSCB destruction under certain pathological conditions (Pedragosa et al., 2018; Santisteban et al., 2020), we investigated whether persistent PVM depletion in SOD1^G93A^ mice is involved in maintaining the physiological function of BSCB. The BSCB destruction was assessed by hemosiderin deposition detected by Prussian blue staining; the number of hemosiderin deposits in the lumbar spinal cord of CLO-treated mice was significantly reduced compared to PBS-treated mice (Figures 5A, B). To gain further insight into BSCB improvement, we examined the extracellular matrix (ECM) that contributes to BSCB maintenance. We observed significantly reduced immunoreactivity of the ECM protein laminin in the lumbar spinal cord of SOD1^G93A^ mice after P120 (early symptomatic stage) (Figures 5C, E). Macrophages can degrade ECM via the production of matrix metalloproteinases (MMPs). PVM depletion reduces MMPs activity in the brain and upregulates ECM-related gene expression in fibroblasts, the main source of ECM, leading to an increase in perivascular ECM (Drieu et al., 2022). Therefore, we investigated the effect of PVM depletion on ECM in SOD1^G93A^ mice and found that laminin immunoreactivity in the lumbar spinal cord of CLO-treated mice was significantly increased compared to PBS-treated mice, indicating that the decrease in laminin with ALS pathological progression was rescued (Figures 5D, E). We also tested another extracellular matrix protein, collagen IV, which has been suggested to increase due to PVM depletion (Drieu et al., 2022). We found that collagen IV immunoreactivity in the lumbar spinal cord of SOD1^G93A^ mice was significantly increased during the disease progression and that of CLO-treated mice was significantly increased compared to PBS-treated mice (Figures 5F, G). These results suggest that PVM depletion in SOD1^G93A^ mice suppresses pathological ECM degradation, maintains the BSCB, and prevents BSCB collapse.

## 4 Discussion

In this study, we investigated whether PVM, immune cells specifically located in the NVU, are involved in ALS pathogenesis. First, we found an increase in CD206^+^ PVM during disease progression in the spinal cord of SOD1^G93A^ mice. We then classified CD206^+^ PVM into anti-inflammatory Lyve1^+^ PVM or pro-inflammatory MHCII^+^ PVM and found that both were increased in the end-stage of SOD1^G93A^ mice compared to WT mice. Interestingly, the ratio of MHCII^+^ PVM to Lyve1^+^ PVM was greater in SOD1^G93A^ mice compared to WT mice, suggesting pro-inflammatory roles. Next, we showed that sustained and selective depletion of PVM by CLO ameliorated the ALS pathology. Finally, we demonstrated that PVM depletion supplemented the extracellular matrix, a component of the BSCB, which was reduced during ALS pathological progression, and prevented BSCB disruption. These results suggest that PVM are an exacerbating factor in the pathogenesis of ALS.

Under steady state, PVM are rarely replenished from circulating monocyte-derived macrophages and persist for a long period of time (Goldmann et al., 2016). In contrast, we showed that PVM increases with disease progression in SOD1^G93A^ mice. There are two possible routes for the increase in PVM. One is an increase due to self-renewal. In the experimental autoimmune encephalomyelitis (EAE) mouse model of multiple sclerosis, it has been suggested that the increase in PVM in the spinal cord during the acute and chronic phases is due to local self-renewal (Goldmann et al., 2016). Another possibility is recruitment of myeloid cells. In ALS patients and animal models, it has been suggested that T cells infiltrate from the periphery to the spinal cord (Chiu et al., 2008; Fiala et al., 2010). It has also been indicated that monocytes infiltrate into the spinal cord (Butovsky et al., 2012), suggesting that the recruited monocytes may have differentiated into PVM. Furthermore, given that recent reports have proposed that skull and vertebral bone marrow directly supply myeloid cells to the CNS compartment (Brioschi et al., 2021; Cugurra et al., 2021), the increase in PVM we observed in the spinal cord of SOD1^G93A^ mice may be due to the recruitment from adjacent vertebral bone marrow-derived macrophages. Future studies should clarify the routes of increased PVM and blocking that pathway may be a therapeutic target for ALS.

Both the anti-inflammatory Lyve1^+^ PVM and pro-inflammatory MHCII^+^ PVM subtypes were increased in SOD1^G93A^ mice compared to WT mice. Furthermore, the MHCII^+^ PVM to Lyve1^+^ PVM ratio was higher in SOD1^G93A^ mice than in WT mice. Increased MHCII^+^ PVM have been observed in the EAE mouse model, suggesting a link between neuroinflammation and EAE pathogenesis (Jeong et al., 2022). A relationship between neuroinflammation and disease progression has also been suggested in ALS (Liu et al., 2021), indicating that PVM shifted to inflammatory cell populations may contribute to the formation of a neuroinflammatory environment. From a literary point of view, in MHCII^+^ PVM, upregulation of proinflammatory genes such as Cxcr4, Il1b, Cxcl9, Cxcl10, Cxcl13, and Cxcl16 has been reported (Jeong et al., 2022). CXCR4/CXCL12 signaling has been reported to be involved in BSCB disruption and loss of tight junctions in SOD1^G93A^ mice (Rabinovich-Nikitin et al., 2016). The inflammatory cytokine IL-1β promotes neuroinflammation and exacerbates the pathology in SOD1^G93A^ mice (Meissner et al., 2010). In addition, chemokines (CXCL9, CXCL10, CXCL13, CXCL16) may be involved in the infiltration of peripheral immune cells into the spinal cord parenchyma observed in SOD1^G93A^ mice (Chiu et al., 2008; Fiala et al., 2010, Butovsky et al., 2012). Thus, the increased MHCII^+^ PVM may be associated with various pathological features of ALS via elevated expression of proinflammatory genes.

We analyzed *in vivo* whether PVM contribute to ALS pathogenesis using a well-established method for sustained PVM depletion with minimally affecting microglia. We found that PVM depletion suppressed motoneuron loss, delayed disease progression, and prolonged survival in SOD1^G93A^ mice. These results indicate that PVM have a detrimental effect on the pathological progression of ALS. We next focused on the association of PVM with the extracellular matrix that maintains BSCB. We observed a decrease in laminin in SOD1^G93A^ mice as the disease progressed, consistent with a previous report (Garbuzova-Davis et al., 2007). Previous reports have suggested that decreases in the extracellular matrix proteins collagen IV and laminin lead to BBB disruption and increased vascular permeability (Wasserman and Schlichter, 2007; Menezes et al., 2014). In our results, before the disease onset (P60), PVM counts and laminin levels in SOD1^G93A^ mice were similar to those in WT mice. However, from P120 onward, laminin levels were decreased inversely to the increase in PVM with disease progression. These results support the possibility that PVM contributes to the decrease in extracellular matrix proteins in SOD1^G93A^ mice. On the other hand, in contrast to laminin, collagen IV levels were increased with disease progression, consistent with the previous report (Miyazaki et al., 2011). They stated that the increase in collagen IV is a compensatory mechanism to prevent BSCB failure, and the present finding that CLO administration increases collagen IV expression may support this mechanism. Our results indicate the possibility that excessive degradation of ECM and reduced BSCB function by PVM may exacerbate ALS pathology and make the motor neurons more vulnerable to damage. It has been reported that impaired BSCB integrity promotes the influx of neurotoxic blood-derived products into the brain parenchyma and exacerbates ALS pathology (Winkler et al., 2014). It has also been reported that PVM contribute to increased vascular permeability in a rat stroke model (Pedragosa et al., 2018) and that Nox2-dependent oxidative stress in PVM impairs the BBB in a mouse model of hypertension (Santisteban et al., 2020).

It is well known that cells within the brain parenchyma such as motor neurons, microglia, and astrocytes are involved in the pathogenesis of ALS (Boillée et al., 2006; Yamanaka et al., 2008). Drug administration to the brain parenchyma is limited by the BBB. Because the PVM reside outside the parenchyma in the CSF-filled perivascular space, drug administration into the CSF avoids the above problems and facilitates drug access to the CNS. With the recent approval of intrathecal treatment of neurodegenerative diseases, it is hoped that new treatments for ALS targeting the PVM can be developed.

This study has three limitations. First, we have not been able to assess the effect of meningeal macrophages on the development of ALS. It is known that CLO injection into the CSF depletes meningeal macrophages as well as PVM (Polfliet et al., 2001). It is undeniable that depletion of meningeal macrophages may also be involved in the amelioration of ALS pathology in SOD1^G93A^ mice. Second, we did not permanently deplete the PVM; given the invasive nature of ALS mice, we stopped CLO injections before the ALS pathology became severe. In the future, genetically engineered mice, such as the CD206^CreERT2^ mouse (Masuda et al., 2022), may be useful to permanently deplete the PVM in SOD1^G93A^ mice and assess its involvement in ALS pathogenesis. Third, only female mice were used in this study. Experiments with both sexes will further strengthen our results.

In summary, our results indicated that PVM was increased, and its properties were altered in SOD1^G93A^ mice during the progression of ALS pathology. Depletion of PVM was also shown to ameliorate ALS pathology by increasing ECM and preventing the destruction of BSCB, suggesting that PVM may be a new therapeutic target for ALS.

## Data availability statement

The raw data supporting the conclusions of this article will be made available by the authors, without undue reservation.

## Ethics statement

The animal study was approved by the Keio University Animal Care and Use Committee. The study was conducted in accordance with the local legislation and institutional requirements.

## Author contributions

KA: Conceptualization, Investigation, Writing – original draft, Writing – review and editing. KM: Investigation, Writing – review and editing. YC: Investigation, Writing – review and editing. MH: Investigation, Writing – review and editing. YM: Investigation, Validation, Writing – review and editing. KY: Methodology, Resources, Writing – review and editing. HM: Conceptualization, Investigation, Writing – original draft.

## Footnotes

1
https://resources.jax.org/therapeutic-area-neurobiology/guide-working-with-als-mice
